# Building bridges between natural and social science disciplines: a standardized methodology to combine data on ecosystem quality trends

**DOI:** 10.1098/rstb.2021.0487

**Published:** 2022-07-04

**Authors:** I. Richter, B. R. Roberts, S. F. Sailley, E. Sullivan, V. V. Cheung, J. Eales, M. Fortnam, J. B. Jontila, C. Maharja, T. Ha. Nguyen, S. Pahl, R. A. Praptiwi, J. Sugardjito, J. D. C. Sumeldan, W. M. Syazwan, A. Y. Then, M. C. Austen

**Affiliations:** ^1^ School of Psychology, University of Plymouth, Plymouth, Devon, UK; ^2^ School of Biological and Marine Sciences, University of Plymouth, Plymouth, Devon, UK; ^3^ Department of Psychology, Norwegian University of Science and Technology, Trondheim, Trøndelag, Norway; ^4^ European Centre for Environment and Human Health, University of Exeter, Exeter, Devon, UK; ^5^ Department of Geography, University of Exeter, Exeter, Devon, UK; ^6^ Plymouth Marine Laboratory, Plymouth, Devon, UK; ^7^ College of Fisheries and Aquatic Sciences, Western Philippines University, Puerto Princesa, Palawan, the Philippines; ^8^ Centre for Sustainable Energy and Resources Management, Universitas Nasional, Jakarta, Indonesia; ^9^ Faculty of Social Work, Hanoi National University of Education, Hanoi, Vietnam; ^10^ Urban and Environmental Psychology Group, University of Vienna, 1010 Vienna, Austria; ^11^ Department of Biotechnology, Universitas Esa Unggul, Jakarta, Indonesia; ^12^ Department of Urban and Regional Planning, Faculty of Built Environment, Universitas Esa Unggul, Jakarta, Indonesia; ^13^ Institute of Biological Sciences, Faculty of Science, Universiti Malaya, Kuala Lumpur, Malaysia; ^14^ Department of Biology, Faculty of Science, Universiti Putra Malaysia, Kuala Lumpur, Malaysia

**Keywords:** interdisciplinarity, marine resources, sustainability, un ocean decade, coastal communities, mapping

## Abstract

Despite a growing interest in interdisciplinary research, systematic ways of how to integrate data from different disciplines are still scarce. We argue that successful resource management relies on two key data sources: natural science data, which represents ecosystem structure and processes, and social science data, which describes people's perceptions and understanding. Both are vital, mutually complementing information sources that can underpin the development of feasible and effective policies and management interventions. To harvest the added value of combined knowledge, a uniform scaling system is needed. In this paper, we propose a standardized methodology to connect and explore different types of quantitative data from the natural and social sciences reflecting temporal trends in ecosystem quality. We demonstrate this methodology with different types of data such as fisheries stocks and mangrove cover on the one hand and community's perceptions on the other. The example data are collected from three United Nations Educational Scientific and Cultural Organization (UNESCO) Biosphere reserves and one marine park in Southeast Asia. To easily identify patterns of convergence or divergence among the datasets, we propose heat maps using colour codes and icons for language- and education-independent understandability. Finally, we discuss the limitations as well as potential implications for resource management and the accompanying communication strategies.

This article is part of the theme issue ‘Nurturing resilient marine ecosystems’.

## 1. Introduction

The 2021–2030 United Nations (UN) Decade of Ocean Science for Sustainable Development aims to facilitate global communication and mutual learning across scientific disciplines and stakeholder communities [1,2]. Allied to this, successful and sustainable marine management requires collaboration among all actors and appropriate concepts, data and information from both the natural and social sciences [3]. Arguably, marine policy to date has been primarily driven by natural science and technocratic approaches, with data derived from stakeholders and communities often considered relatively weak, inferior or incomplete by both policy makers and natural scientists [4–6]. This is despite approaches such as marine spatial planning being adopted with a view to increase stakeholder participation [7]. The UN Ocean Decade encourages a transformative approach in ocean science, spanning multiple disciplines by actively integrating natural and social sciences and embracing local and indigenous knowledge as a key knowledge source [2,8]. The UN Ocean Decade explicitly calls for co-design, inclusion of all appropriate concepts, and regional action and encourages the scientific community to step away from producing more knowledge and instead to build connections and dialogue between different science disciplines. Yet, when different types of data have not been purposefully collected with the objective to subsequently bring them together, it can be a challenge to combine and compare them. There is a need for methodologies that enable the systematic coordination and integration of a broad variety of knowledge systems and their communalities and differences. There is also a need to communicate such knowledge appropriately to practitioners for sustainable and inclusive marine resource management and improved engagement.

This opinion piece presents a standardized methodology to integrate natural and social science data. For demonstration purposes, we use data collected within the Global Challenges Research Fund (GCRF) Blue Communities Project, representing quality trajectories in fisheries as well as mangrove, coral and seagrass ecosystems in four Southeast Asian locations. We hope to inspire other scholars to further develop this methodological proposal and jointly develop strategies on how information from different knowledge traditions can be combined for the benefit of the bigger picture.

### (a) Previous examples of cross-disciplinary data integration

Increasing volumes of empirical work have shown that combining data from different disciplines has the potential to build a clearer picture of current and future ecological scenarios through complementary processes [9,10].

Combining knowledge sources might lead to more confidence and depth of information. So can a biological measure of fish abundance indicate trends over the last decade, but is limited in explaining why fish stocks have changed over the last century (e.g. the decline of the North Sea cod [11]). Additional data from the natural sciences (e.g. on climate change) or the social sciences such as historical research or interviews with community members, fishers and divers can provide different perspectives and potential reasons for observed changes in fish stock abundance such as the introduction of scallop dredging in the area [12], or increasing numbers of private yachts [13].

An example where different types of data were purposefully combined comes from Cu Lao Cham-Hoi An Biosphere Reserve, Vietnam, where quantitative analyses of spatial and temporal variability in the state of mangrove and seagrass habitats retrieved from satellite imagery are complemented by qualitative community perception data to gain a deeper understanding of the drivers of change [14,15]. This combination of data sources enabled the researchers to identify reasons for mangrove degradation over the previous four centuries, and thereby could feed into evidence-based management policies. Another example comes from Torrents-Tico *et al*. [16]. They integrated estimations of abundances of threatened carnivores of both, indiginous and local knowledge, as well as monitoring data. The researchers found that the types of data both converge and diverge. They interpret this finding as that divergences show limitations in scientific sampling methods, and that an understanding of socio-psychological and cultural influences is indispensable for effective conservation efforts. Other examples are provided by Laidler [17] on sea ice abundance, by Moller *et al*. [18] on wildlife harvest and by Mackinson [19] on fisheries stocks.

Another potential benefit of integrating natural and social science data might be that resulting resource management plans may be more appropriate for, and accepted by, the local communities [20]. Less than half of coastal and marine planning processes includes social data, and only 10.8% of social data are analysed spatially, according to a review by Le Cornu *et al*. [21]. Evidence gained through quantitative community perception data derived from large samples, especially representing communities located in the Global South, is yet particularly scarce.

### (b) Lack of standardized methodologies

To date, there is no agreed method or best practice for the integration of different types of data [22]. Previous studies that have attempted such integrations demonstrated the benefits, but also the challenges of doing so [23].

In their meta-analysis across 47 articles and chapters on integrating local ecological knowledge with natural science derived knowledge, Bohensky & Maru [24] conclude that appropriate frameworks are still missing on how to integrate, understand and communicate the complementary knowledge systems. Integration efforts are further complicated by different types of data being used even within disciplines. For example, social science data can range from qualitative (e.g. consisting of spoken and recorded language [25]) to quantitative (e.g. ratings on standardized response scales [26]). While the inclusion of several disciplines provides added value for conservation [10], it also increases the complexity of the research [23]. Therefore, standardized methodologies for comparing social and natural science data should be developed and evaluated [27].

### (c) Discipline-specific biases and challenges to knowledge integration

Integrating different knowledge systems may support a more rounded understanding of the drivers behind changes in environmental state [10]. We need to recognize, however, the discipline-specific ways of drawing conclusions and limitations [28]. Respecting and valuing different perspectives can facilitate a comprehensive understanding of environmental phenomena and can support the development of tailor-made ideas for conservation (for examples see Cottet *et al*. [29], Ioana-Toroimac *et al*. [30] or Foale [31]).

#### (i) Data limitations in the social sciences

Data collected from communities has the benefit of coming from a large group of people who observe their environment all year round. Community members have traditional ecological knowledge developed over generations and based on first-hand experiences [32]. However, by its very nature, this kind of knowledge captures primarily subjective perceptions of the world and relies on self-reports collected through survey questionnaires, interviews, focus groups, observations or combinations thereof [33]. Each of these methods comes with its own strengths and shortcomings, making community perceptions data prone to a range of biases.

To illustrate these biases, Gifford *et al*. [34] collated perceptions of general indicators of ecological quality and temporal trends, including future trends across 18 nations from a large sample of *n* = 3232 respondents. The authors state that community perceptions provided valuable insights and that they correlated closely with expert ratings of ecological quality. However, they also point to biases such as temporal pessimism and spatial optimism, potentially preventing sustainable behaviour change. On a similar note, Jung *et al*. [12], who combined retrospective stakeholder perceptions with biological measurements, recognized the role of demographic factors in explaining variance between respondents. Further, Parson *et al*. [13] note that most respondents do not refer to time periods greater than 10 years ago, which is also in line with the recommendation for appropriate ‘human time horizons’ for effective communication by Fincher *et al*. [35]. Another phenomenon affecting reports of environmental change is the Shifting Baselines Syndrome (SBS, [36]), indicating that younger generations adapt to their current environment, including the level of degradation, as their norm, resulting in inaccurate baselines being taken as the starting point. Community perceptions are additionally prone to be influenced by significant events, such as an oil spill, or salient environmental changes such as the removal of trees, which have occurred recently or close to the local vicinity. These influencing factors can account for some of the differences within and between informant groups [37].

All these cognitive biases need to be considered when using, interpreting and communicating perception data as an information source for environmental quality. It is also not only the people providing the perceptions data who may potentially be influenced by cognitive biases, and recent events, but equally those who use these data, such as policy makers and stakeholders.

#### (ii) Data limitations in the natural sciences

Similar to data in the social sciences, natural science data varies in its nature and can range from physical (e.g. ocean currents, temperature), biogeochemical (e.g. nutrient) to biological (e.g. productivity, biomass of specific groups). Each type of data is typically measured with maximum objectivity and repeatability at different spatio-temporal scales. Sources of natural science data for environmental monitoring can include *in situ* sampling (periodic sampling or long-term time series at specific sites such as the Western Channel Observatory (https://www.westernchannelobservatory.org.uk/), remote sensing observations from satellites to map land cover or vegetation, and marine system models that expand the spatio-temporal range of the data. These data sources can provide information about natural variations in the ecosystem state (e.g. seasonal, annual and decadal as described in Doney & Sailley [38]) as well as indicate change in the state of the system over time (i.e. improvement or degradation as described in Hsieh *et al*. [39]).

Natural science data may also be subjected to biases with different methodologies having their own limitations [40]. Biological sampling may be undertaken at individual study sites with periodic measurements, capturing a snapshot of the ecosystem state at these locations at specific points in time (e.g. data collection in Antarctica is mostly undertaken during the austral summer months to take advantage of the ice free conditions). The frequency or timing of the sampling may be influenced by practical considerations, such as site accessibility, available time in the field, funding and seasonal weather patterns [41]. In a study by Johannes *et al*. [42], scientists conducted their research during the day not knowing that the fish species of interest was active at night—resulting in incorrect conclusions being drawn. Similarly, remote sensing approaches for environmental monitoring have limitations in terms of observation period and temporal resolution, as the revisit time and frequency of observations will depend on the satellite's orbit, the location of interest and sensor swath, as well as local conditions such as cloud cover if optical imagery is used [43]. Satellites or aircrafts typically provide a snapshot of the feature of interest from above, which needs to be considered when drawing conclusions. Optical imagery over a forest, for example, reflects the forest canopy; and infrared sea surface temperature measurements reflect the conditions in the upper 10 µm of the water column.

Finally, multiple types of marine system models exist, assessing either the physical, biogeochemical or biological processes [44–46]. As a result, the comparison of such models is a complex field of its own [47,48]. Typical examples of interactions between the different types of natural science data include the validation of remote sensing through ground truthing surveys via *in situ* sampling, assimilation of data from either sampling or remote sensing into models [49] and interactions between time series and models for validation or development of new paradigms [50]. These projects strengthen the argument that different types of data make different contributions, and that their combination provides important added value [51].

#### (iii) Pathways to knowledge integration

Owing to the aforementioned limitations, relying exclusively on one data source might run the risk of having incomplete or skewed conclusions. Bringing together several knowledge systems can lead to a mutual filling of gaps and an overall strengthening of comprehensive datasets [20]. It is, however, important to carefully consider the characteristics and limitations of each data source as well as the simplifications required for integration. Challenges for data integration include temporal or spatial scale differences [52,53], different terminologies between disciplines and different strategies to interpret the same content [9,54]. Several reviews and commentaries highlight the need for respect and sensitivity in interdisciplinary work that includes community-derived knowledge (e.g. [18,55,56]). Key recommendations are for scientists to engage in synergy-building and culturally appropriate interactions that promote trust and openness, equitable and respectful partnerships, co-production of knowledge and common visions [57].

### (d) The current approach and study sites

**Figure 1.**
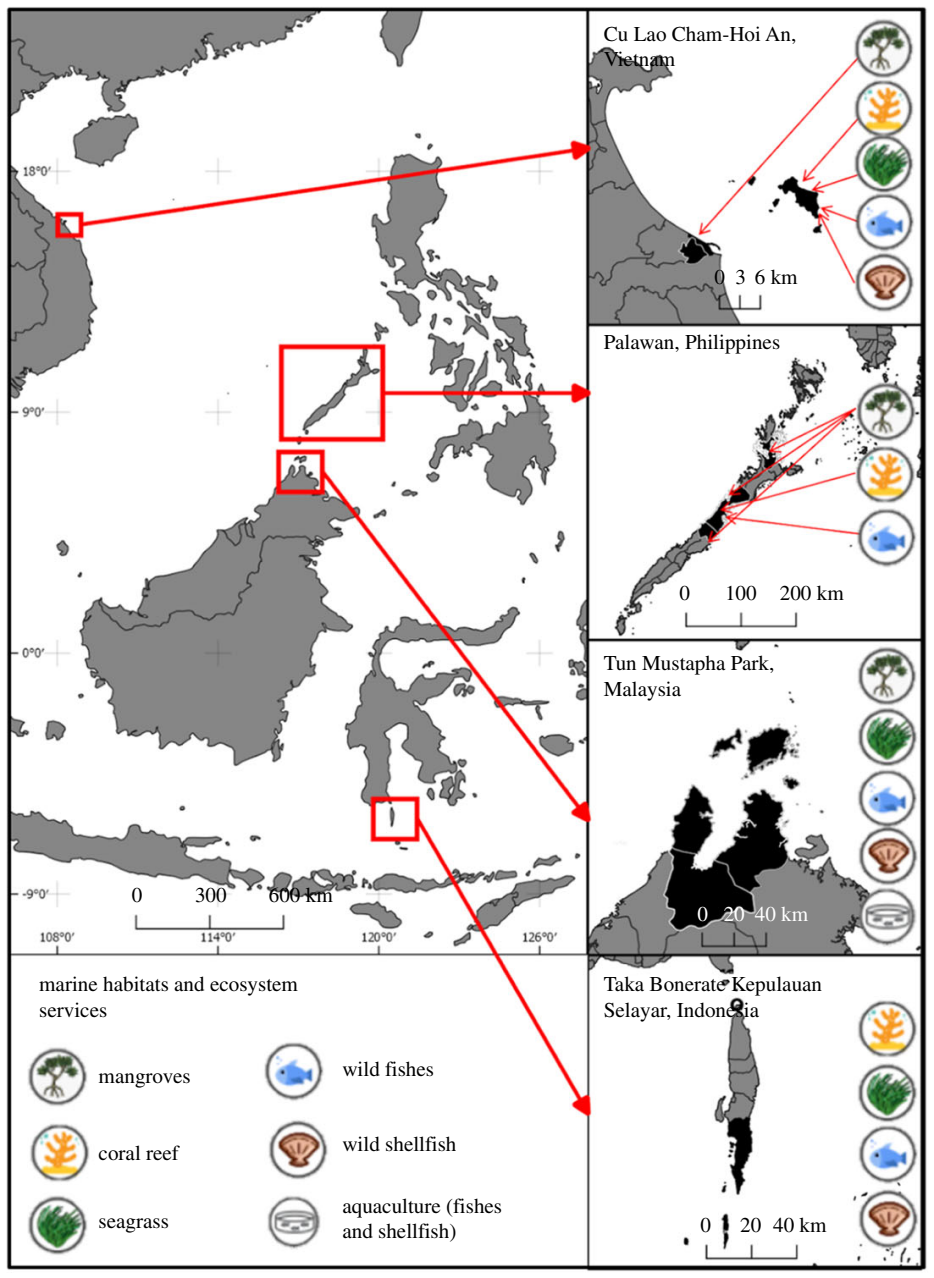
Habitats and ecosystems included for data integration for four South East Asian case study sites. Coastal communities participating in this study are located within these sites (as described in the section on study data). The different icons represent the type of data included per site.

In this opinion piece, we address the need for developing methodologies to enable the systematic coordination and integration of different knowledge systems for sustainable resource management. We propose a standardized methodology to combine and communicate quantitative data on temporal trends of marine ecosystem quality from the natural and the social sciences, selecting fisheries, mangroves, corals and seagrass as examples. Using four case study sites (1–4, see below) from the GCRF Blue Communities Programme (www.blue-communities.org), we demonstrate how different types of quantitative ecosystem data can be brought together and mapped systematically to reflect trends of past and future coastal ecosystem quality (see figure 1 for an overview of the different types of data and the locations of the study sites).

By developing a standardized method of mapping these different types of data, we can identify corresponding and disparate trends between datasets. We suggest heat maps and icons to visually represent these trends and allow for relatively literacy-independent understanding. We also discuss opportunities of how these maps can be used in sustainable resource management and point out the limitations of our approach.

Unique assets of our approach are the direct involvement of coastal communities as knowledge contributors, the multitude of researchers from different geographical areas, the range of scientific disciplines (psychology, ecosystem modelling, fisheries and earth observation) and ecosystems as well as the coverage of both the past and the future.

The four study sites namely, (1) Cu Lao Cham-Hoi An United Nations Educational, Scientific and Cultural Organization (UNESCO) Biosphere Reserve (Vietnam), (2) Palawan UNESCO Biosphere Reserve (the Philippines), (3) Taka Bonerate Kepulauan Selayar UNESCO Biosphere Reserve (Indonesia) and (4) Tun Mustapha Park Marine Protected Area (MPA; in Sabah, Malaysia) are located in Southeast Asia (figure 1). All sites are home to important ecosystems such as coral reefs, seagrass beds and mangrove forest habitats that serve as breeding and nursery grounds for a diversity of resident marine species, as well as migratory species such as whale sharks.

(1) Cu Lao Cham-Hoi An was designated as a Biosphere Reserve by the UNESCO Man and the Biosphere (MAB) in 2009 and features unique characteristics and values [58]. The core area of Cu Lao Cham – Hoi An Biosphere Reserve is an MPA which is surrounded by a buffer zone. Particularly important habitats in the area are seagrass beds, mangrove forests and coral reefs [14,15,58]. These ecosystems provide nurseries for wild fish and shellfish, which are central sources of food for the local population.
(2) Palawan is the largest province in the Philippine archipelago composed of one main island and more than 1700 smaller islands [59]. It is known as the country's ‘last ecological frontier' owing to its high biodiversity (35%) and endemicity (5%), and vast terrestrial and mangrove forests that comprise 48% of the country's total forest. Recognizing the outstanding value of rich biodiversity and culture in Palawan, it was declared an MAB Reserve by UNESCO in 1990. Habitats and ecosystems services of critical importance included in this study are the coral reefs, wild fish and mangrove forest.
(3) Designated in 2015, Taka Bonerate Kepulauan Selayar Biosphere Reserve is located in Selayar Islands Regency, South Sulawesi, Indonesia. The estimated population in the area is 134 280 (in 2018) and spreads across approximately 130 islands (total terrestrial area of 1357 km^2^) [60]. The reserve hosts the largest atoll in Southeast Asia, with significant habitats including wild fish stocks, coral reefs and mangroves [61].
(4) Tun Mustapha Park (TMP) in the Malaysian state of Sabah, located at the northern part of the island of Borneo, is the largest multiple-use marine park in Malaysia. Gazetted in 2016, the area covers 898 762.8 hectares with more than 50 islands and islets within the Coral Triangle region [62]. A key habitat in TMP is mangrove forest and the area also contributes more than 12% of Sabah's marine fisheries and aquaculture production [63].

### (e) Standardization methodology

Throughout the 4-year GCRF Blue Communities project, a wide range of natural and social science data has been collected and collated from existing studies covering topics such as fishing and aquaculture, mangroves, corals and seagrass support sustainable interactions with marine ecosystems [64]. Data characteristics and associated data collection methods are succinctly described below, and with more details in the electronic supplementary material.

To be able to map and compare the different data types they have to be standardized. In a generalized manner of speaking, we transform all data into a single value that is comparable independent of the shape and size of the dataset. The social science data were represented with two data points (perceived trends of past and future), while the natural science dataset was represented through time series. The natural and social science data were matched over corresponding 10-year periods. Now the methodology can be split into two strands: (i) the social science data were measured on either a 5- or 7-point Likert scale. The 7-point scales have been converted to a 5-point scale, giving us five categories (strong improvement indicated, improvement indicated, no or slight change, decline indicated, strong decline indicated). The median value of these scales was chosen as a comparison score; and (ii) for the natural science data, the year-to-year change in percentage for each site has been calculated across the entire time series. Subsequently, we determined a cut-off point for each category using one of the two following approaches: (a) a significant value identified from the literature (coral, seagrass, mangrove), or (b) if no such value existed, the median change across all sites to determine a cut-off point value (fisheries). With this approach, all data were transformed into a singular data point that covered a specific time-period. The cut-off point had to be identified for each specific dataset to allow for the amplitude of change in different sectors (e.g. changes in mangrove are less than 5% while those in fisheries can be 50% or more).

The specific approach for each dataset is detailed below. Harmonized scores for all sites covering both present and future data, can be seen in table 1. All the scoring results, as well as the raw data, are presented in the electronic supplementary material.

**Table 1.** Category values for each sector of the natural science (NS) data and the community perception data (CP), the meaning of the perceived or calculated change harmonized to a 5-point scale with both numerical value and arrows for visualization.

fisheries year-on-year median change (%)	mangrove annual rate of mangrove extent change (%)	coral annual rate of coral extent change (%)	seagrass annual rate of seagrass beds extent change (%)	community perception original 5-point/7-point Likert scale values	meaning	5 point scale	arrows for NS (red) and CP (blue)
>20	>0.5	>2	>5	2/3	strong improvement indicated	+2	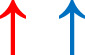
>10 to 20	>0.1 to 0.5	>1 to 2	>1 to 5	1 / 1, 2	improvement indicated	+1	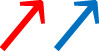
−10 to 10	−0.1 to 0.1	−1 to 1	−1 to 1	0	no or slight change indicated	0	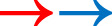
<−10 to −20	<−0.1 to −0.5	<−1 to 2	<−1 to −5	−1 / −1, −2	decline indicated	−1	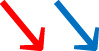
<−20	<−0.5	<2	<5	−2 / −3	strong decline indicated	−2	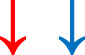

#### (i) Fisheries: present and future

Fisheries and aquaculture data for the last decade was obtained individually for each case study site through official surveys. The annual fisheries statistics for Puerto Princesa City comes from Puerto Princesa City Agriculture Office, for Selayar from the Selayar Bureau of Statistics in Indonesia and for TMP districts from the Department of Fisheries Sabah in Malaysia. The datasets represent different approximations to fish stock status (that is their biomass) either through measuring the size of the wild fish population through surveys (stock surveys, that is research cruise with the explicit goal of estimating the fish stock size) or by recording fish landings for either aquaculture or wild fisheries. These were complemented by model projections of key fish species biomass (10.5281/zenodo.4281146) to provide future data for the next decade.

We included either fish landings data (Indonesia, Malaysia) or stock surveys (Philippines). To match the socio-economic background of respondents who mainly depended on artisanal fisheries, only wild catches landed by traditional fishing gears were analysed [63,65]. Considering the risk that the beginning or end of the time series could be outliers and therefore bias the outcome, an average year-on-year change was calculated and expressed as percentage of change. Like this, the measured year-on-year change varied from +25% to −50% with a median value of +2% and a standard deviation of 18. We used this to create categories to scale the natural science data to the 5-point scale required for comparison (table 1). In relation to the overall value of change across the 10-year period, this method produces a similar value and overall direction of change which is less affected by extremes. The same method was applied to the model outputs for the future data. To make sure that slightly different prospects of climate forcing do not cause significant differences in the final scoring, two popular representative concentration pathways (RCPs) have been calculated and compared. No significant differences have been found and the same final score was obtained for both RCPs. We found that the measured change from year to year varied between +/− 15%, which is within the natural variability found in the present-day data. The median change per site and scenario varied from −5% to +1% which represents a slight change. We are aware of the potential bias for high variability within fisheries data owing to either natural variability in the fish populations (e.g. small pelagic stocks like sardines and anchovies) or changes in the fishing effort itself.

#### (ii) Mangrove data

Mangrove forest areas for study sites in Malaysia, Indonesia and the Philippines were extracted from the Global Mangrove Watch (GMW) dataset [66]. This global-scale dataset is generated from the classification of optical and radar satellite data. For further details on the remote sensing methodology and accuracy assessment, see Bunting *et al*. [66]. Data were available for the years 2007–2010 and 2015–2016. It is anticipated that the accuracy will vary between locations owing to local conditions such as the surrounding vegetation, mangrove condition and satellite data availability. To correspond to the community perceptions data, aggregated at a district level, mangrove extent was extracted for the respective relevant districts or municipality areas. For Cu Lao Cham-Hoi An Biosphere Reserve, mangrove extent for the years 2003 and 2016 was obtained from a study by Tin *et al*. [14]. The mangrove extent estimates in this study were generated using supervised classification of optical Landsat imagery. For further details on the remote sensing methodology and accuracy assessment, see Tin *et al*. [14].

For each site, the mean annual rate of change in mangrove cover was calculated for the time available. The mean annual rate of change was used because the estimates from different sources were available for different time periods and change was expressed as a percentage, rather than net change. The mean annual rate of change in mangrove extent at the study sites ranged from −0.03% to −13.45%, with a median rate of −0.16%. To compare the direction of the trends shown in the mangrove extent data with the community perception of mangrove condition, the data were scaled to a 5-point scale, as above. To determine reasonable thresholds for this ecosystem type, the literature was reviewed to find recent estimates of rates of mangrove change in this region. Estimates of national mangrove deforestation rates ranged from 0.0 to −0.5% per year for the periods 2000–2012 and 2000–2016 for countries in Southeast Asia [67,68]. The median (0.1%) and maximum (0.5%) national rates of mangrove deforestation identified in these studies were used to define the category boundaries. The spatial data from GMW for the study sites were reviewed to confirm this scale was appropriate. The resulting scores assume that a net increase in mangrove spatial extent reflects an improvement in mangrove status and a decrease in extent reflects a decline. These data do not directly reflect comprehensive ecosystem health or condition of mangrove stands but the geographical area covered by mangrove canopy only, which we use as an indicator for mangrove ecosystem quality.

#### (iii) Coral data

For Cu Lao Cham-Hoi An, coral data were obtained from the management board of Cu Lao Cham MPA [58]. Annually, the reef check method is used to observe the area around Cu Lao Cham MPA, providing data on coral reef cover. In Palawan, time series data for corals (1997–2017) is available for Puerto Princesa only [69]. Owing to gaps in the dataset, some values had to be interpolated. Different methods were used including the line intercept method [70], point intercept transect [71] and reef check method [72]. Only the percentage of hard coral cover is included in this study. For Selayar, two Sentinel-2 satellite images for the years of 2015 and 2019 were obtained from an open access European ESA-Copernicus project portal (https://scihub.copernicus.eu/). The Sentinel-2 product includes orthorectified top-of-atmosphere (TOA) reflectance (Level 1-C). This version also contains sub-pixel multispectral registration. For the habitat classification related to coral platforms, we focused the analyses on two sites, Tambolongan and Polassi islands, which were selected as representative for the region (Bontosikuyu). Subsequent to this, unsupervised isodata classification was conducted using the following criteria: a maximum of 20 classes on Sentinel-2 using bands 2–3–4–8 with 10 m of resolution [73]. Output images were obtained, from which we derived classes for each pixel area, to calculate coral reef coverage using QGIS. The assignment was based on interpreting the geomorphologic description of the seabed, with particular reference for coral identifications [74].

To create a 5-point scale and define valid categories (as shown in table 1), the mean values of the change in live coral reef cover from 2011 to 2021 was used as the value signifying average yearly change rate in Cu Lao Cham-Hoi An and the per cent change in the mean values of hard coral cover between 2007 and 2017 was computed in Palawan. For Indonesia, two datasets were available, and the mean rates of change were calculated for 2015 and 2019. To cross-validate, Bruno & Selig [75] estimated yearly coral cover loss in the Indo-Pacific area was approximately 1% over the last 20 years and 2% between 1997 and 2003 (equal to 3168 km^2^ yr^−1^). These data do not directly reflect comprehensive ecosystem health or condition of coral reefs, but the geographical area covered by coral only, which we use as an indicator for coral ecosystem quality.

#### (iv) Seagrass data

Seagrass extent data could only be found for one study site, namely Cu Lao Cham-Hoi An. The extent of data of seagrass beds in 2003, 2010 and 2017 was obtained from Tin *et al*. [15]. The authors used ALOS AVNIR-2 and Landsat satellite imagery and field surveys to map the area of seagrass beds in the selected years.

The mean rate of change in seagrass bed cover including unchanged, lost and gained areas from 2003 to 2017 in Cu Lao Cham was calculated. In a global analysis on temporal changes in seagrass beds, Waycott *et al*. [76] estimated that seagrass beds were disappearing at a rate of 7% year globally. In a literature review from 2000 to 2020, an overall average decline of seagrass beds of 4.7% per year was identified [77]. As no literature was available on average changes of seagrass in Vietnam specifically and the maximum decline measured in the literature was 4.7%, a rate of 5% was used as a guide to compare the change in seagrass beds extent to the community perception data (table 1). These data do not directly reflect comprehensive ecosystem health or condition of seagrass beds, but the geographical area covered by seagrass only, which we use as an indicator for seagrass ecosystem quality.

#### (v) Community perception data

To assess perceptions of the quality of the marine environment, a survey was co-developed with local communities in each case study site. The questions aimed to understand people's past and future perceptions of marine quality. An example question assessing perceived past quality of wild fish catch in the Philippine case study site is: ‘Compared to 10 years ago, would you say the current amount of wild fish and diversity of fish types in Palawan is better, worse or the same?’. To assess future perceptions, the wording was: ‘Compared to now, how do you see the amount of wild fish and diversity of fish types in Palawan in the future in 10 years' time.' Data were collected from coastal communities in the following locations: in Palawan, Philippines data were collected in Aborlan, Taytay and Puerto Princesa; in Sabah, Malaysia data were collected in Kudat, Kota Marudu and Pitas; in Hoi An-Cu Lao Cham, Vietnam data were collected at two mainland coastal communities in Hoi An: Cua Dai and Cam Thanh, and in Tan Hiep on the Cu Lao Cham island; in Taka Bonerate Kepulauan Selayar, Indonesia data were collected in Bontosikuyu. Sample sizes for each case study were *n* = 252 in Vietnam, *n* = 401 in the Philippines, *n* = 330 in Indonesia and *n*= 610 in Malaysia (representing approximately 10% of respective local communities) with varying response rates for different items, depending on professional expertise or location of residence (electronic supplementary material, table S15). Demographic profiles vary across case study sites and are not fully representative of the respective populations in terms of age or gender. This is mainly owing to subsistence-based restrictions such as middmen having to follow their daily routines while women stayed at home and have been available for data collections.

Perceptions about the past and future marine environmental quality were captured on either a 7-point (from −3 to 3 in the Philippines, Malaysia and Vietnam) or 5-point (from −2 to 2 in Indonesia) Likert scale ranging from ‘much worse' (maximum negative score) over ‘no change perceived' (zero) to ‘much better' (maximum positive score). The individual decision on the range of the Likert scale was made by local experts in each country, resulting in the intra-project variance. Full details of how data were harmonized between countries, as well as the data collection dates and demographic details, can be found in the electronic supplementary material.

### (f) Methodology for harmonizing and mapping the scores

**Figure 2.**
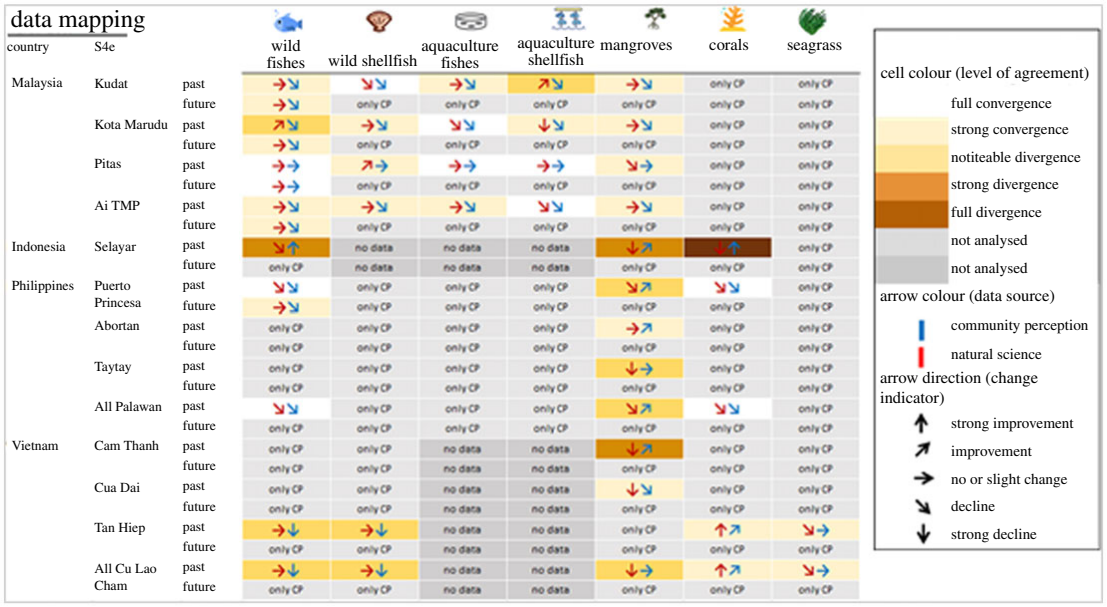
Colour coded heat map illustrating scores of convergence and divergence across sites, but also including arrows of different levels of steepness representing trends for habitat quality.

By harmonizing all natural and social science data to a 5-point scale ranging from −2 to 2, and then subtracting these scores from each other (in specific, we subtract the community perception data (CP) score from the natural science (NS) score), we could acquire a new set of scores (ranging from −4 to +4) that indicates how closely the two types of data overlap. This gives us three possibilities: (i) the scoring of the CP and NS has the same value (e.g. both have a −2 or a +1 score), this will result in a final score of 0 once substracted, indicating full convergence; (ii) if the scores for CP and NS are close to each other we will have a value around 1 or 2 (e.g. a score of −2 and −1 for NS and CP, respectively, will give a final score of −1), indicating string convergence; and (iii) the scores are opposites, indicating a noticeable or strong divergence in the scores (e.g. a score of +2 and −2 for NS and CP, respectively, will give a final score of +4). The positive values (1, 2, 3, 4) hereby represent cases in which the natural sciences indicate a more positive trend than the social sciences, whereas the negative values (−1, −2, −3, −4) indicate cases in which the social sciences indicate a more positive trend than the natural sciences. We used a range of colour scales to represent these scores, with lighter colours indicating a higher level of convergence between social and natural sciences data (figure 2 for final scores and legend).

## 2. Results

We present the temporal trends followed by an elaboration on the results by case study area and sites in the discussion.

Figure 2 illustrates details on the trends represented by the natural and the social sciences (illustrated with coloured arrows, red to represent NS, blue to represent CP), the level of convergence and divergence (colour code of cells and direction of arrows, table 1) as well as icons for habitats for intuitive understanding (figure 1). We developed this way of visualization as it allows easy interpretation of our results by a broad audience, independent of literacy and language skills. This is particularly relevant as we collated data from four different case study sites, across four countries and covering remote areas with relatively low rates of general literacy. For the basic heat map representing scores of convergence and divergence ranging from 0 (full convergence) to −4/+4 (full divergence), see figure S1 in the electronic supplementary material.

## 3. Discussion

In the spirit of the UN Ocean Decade, this work brings together different forms of knowledge to feed into inclusive and localized policy making. We propose a systematic methodology for comprehensive and objective data integration. The data included is of quantitative nature and comes from both the natural and the social sciences.

The results produced with this methodology are visualized in a heat map in which areas of convergence and divergence between the natural sciences and community perceptions can be identified for each case study area.

The different levels of convergence and divergence can be used as a starting point for discussions around potential causes, future co-development of resource management plans, and the evaluation of existing resource conservation programmes.

In the following section, we demonstrate how the results can be interpreted. In each of our case study sites, the mapping exercise led to a unique profile of convergence and divergence, which will be discussed individually and then set into perspective with past and current local resource management.

### (a) Cu Lao Cham-Hoi An biosphere reserve, Vietnam

In Cu Lao Cham-Hoi An, a high overall level of convergence between natural and social sciences was observed. Data on coral reefs and seagrass beds from the natural and social sciences represent similar or equal, improving (coral) or declining/stable (seagrass) trends.

A potential reason for this high level of agreement over coral reef cover might be the community-inclusive annual reef check and coral clean-up programmes which have been running since 2011 [58]. These programmes are not only aimed at cultivating and restoring the coral reefs, but also at applying co-management models which promote the participation of the local communities into policy implementation. Community involvement in conservation efforts can influence people's understanding of their local marine environments [78]. For seagrass cover, the natural science data, which integrated remote sensing imagery, GIS technology and *in situ* field studies, indicates that the area of seagrass beds have declined rapidly and have almost disappeared in Cu Lao Cham MPA [15]. According to Tin *et al*. [15], the pollution of the marine environment and destructive fishing methods are major reasons contributing to these declines. However, community perceptions on seagrass beds indicate no change over the last 10 years. This might be owing to seagrass being an invisible ecosystem because of its submerged location under the ocean surface or poor media coverage. Compared to coral reefs and mangroves, seagrass beds have not been part of any campaign or public conservation effort in Cu Lao Cham, Hoi An. Targeted communication around the importance of seagrass beds might be a possibility to engage communities in the protection of this habitat.

The habitat that showed the highest levels of divergence was mangrove forests, especially in Cam Thanh, where we found opposite trends (strong divergence) for the natural sciences (degrading) and community perceptions (strongly improving). In Cam Thanh (a site on the mainland), mangroves were perceived to improve by the communities whereas the natural sciences indicated a strong decline. An explanation for this disagreement could be that citizens of Cam Thanh are using nipa palm as a building material and are therefore motivated to preserve this mangrove species. Nipa palm trees are subject of targeted, successful conservation efforts, whereas other mangrove species or the forest as a whole might be undergoing significant decline. From 2010 to 2013, Cam Thanh Communal Association of Farmers implemented a project called ‘Rehabilitation and conservation of nyapalms [sic] at Cam Thanh Commune, Hoi An City, Quang Nam’ in order to protect land at the Thu Bon river from erosion and thereby contribute to nipa palm conservation [79]. This successful conservation effort, albeit selective, might lead to communities perceiving their mangroves being in a good state, even if the overall mangrove cover might decline as indicated by the natural science measurements. Additional data-related biases could be that the area covered by the natural science data are larger in terms of km^2^ than the areas covered by the community surveys. Secondly, the natural science data for mangroves are from two snapshots in time only (2003 and 2016) which may not capture more recent conservation efforts or younger mangrove stands.

### (b) Palawan, Philippines

In Palawan, a variety of convergence and divergence levels were observed between the two knowledge sources. In Puerto Princesa City, the community perception and natural science data were in full convergence for past wild fish stocks and strong convergence for coral reefs, with trends indicating that wild fish stocks have declined, and coral reefs have slightly degraded over the last 10 years. Illegal and destructive fishing methods were a common problem in the past (during the 1990s) in Palawan, causing not only the decline of corals and fish stocks but also gaining media attention and thereby influencing community perceptions. Although there are efforts to establish MPAs, the communities expected the wild fish to further decline in the future, whereas the natural sciences predicted a stable trend. This negative prediction of the communities might be owing to the noticeable increase of visitor numbers, especially in Puerto Princesa (the island's capital and location of the airport), which will most likely lead to a surge in demand for seafood.

Community perceptions and natural science measurements in Palawan noticeably diverged for mangroves. In all three municipalities, the communities perceived the mangroves to be in a better state than the natural science measurements suggested, which is similar to the pattern found in Vietnam. In Taytay, communities perceived no change in the mangrove forests, compared to the natural science data, which indicated a decrease over the past 10 years. The natural science data from GMW showed mangrove loss in Taytay was diffuse and primarily on coastal fringes and river edges, areas that are particularly challenging to monitor owing to their geographical dynamics and potentially not visible to the communities [66]. In Aborlan and Puerto Princesa, communities perceived mangrove forests to expand while the natural sciences reported either a decline or stable condition. The reason for this could be found in the popularity of Palawan as a Mangrove Swamp Forest Reserve since 1981 (PPD 2152). Mangrove planting and reforestation initiatives are a popular social activity on the island, extensively covered by the media; in Puerto Princesa, for example, 800 000 mangroves have been planted since 2003 (https://puertoprincesa.ph/?q=tourism/february-14-love-affair-nature). These very salient and community-inclusive initiatives potentially lead to the positive perceptions of mangrove health within the population despite severe mangrove deforestation for domestic and industrial usage [80]. It is also likely that the natural science data will not detect the newly planted mangroves until they are relatively mature, as the remote sensing technique uses image classification to map mangrove vegetation cover, and when trees are young they may be too small to be detected relative to the surrounding water and bare ground signals within a pixel, particularly at lower resolution.

### (c) Taka Bonerate Kepulauan Selayar Biosphere Reserve, Indonesia

In Selayar, the community perception and natural science data appeared to diverge strongly across all areas (corals, mangroves and fish catch) with communities having more positive perceptions compared to the natural science data. Potential reasons could be that policy makers on the island strongly prioritize the promotion of the island as an attractive tourist destination and thereby boosting the economy.

There are some participatory resource management programmes that have been conducted in the area such as Coremap 1 and 2 (1998–2009), and the Capturing Coral Reef & Related Ecosystem Services (CCRES) project (2014–2018). Several outputs produced from those programmes include the participatory formation of MPAs, increased capacity of local people in marine management, and production of numerous technical tools to assist local stakeholders in marine management [81,82]. However, despite these efforts, degradation of crucial marine habitats, such as coral reefs, are still rampant. For instance, the practice of destructive fishing and extraction of corals for building materials are deemed to be persistent problems in the area [83,84]. Kusumo *et al*. [85] estimated that in Selayar's coastal waters, damaged reef areas increase by the rate of approximately 10–50 m^2^ d^−1^ owing to cyanide fishing. These problems were exacerbated by other factors, such as increasing numbers of the anchoring of tourist boats [86].

This dichotomy of overly positive reporting about the island's beauty alongside ongoing destructive fishing practices could explain the high levels of divergence we observe between the natural science and community perceptions in Selayar. Responsibility diffusion and the shifting baseline syndrome may underlie this divergence in addition. A recent study in Selayar, conducted in 2018 [87], indicated that coastal communities in the region had positive perceptions of their local coral reefs, and tended to consider ‘other communities' to be responsible for environmental degradation happening in the island. By ascribing the responsibility for environmental decline to others, people can maintain the collective values within their own community [88]. An additional reason could be that people's perceptions adapt to the gradual degradation of their natural environment as the new status quo and they do not interpret it as problematic [89].

### (d) Tun Mustapha Park, Malaysia

In TMP, community perceptions and natural science data converged across most areas, although divergences were observed for the state of fisheries at the district level. In most cases, community perceptions were less positive than the natural science data. Natural science data for wild fish landings in Kota Marudu and shellfish aquaculture productivity in Kudat showed an increase over the last 10 years but were perceived to be declining by the communities. One potential reason for this divergence could stem from the demographic profile of participants. Only 17% of respondents directly worked in the fisheries and aquaculture sector potentially leading to inaccurate perceptions (see the electronic supplementary material, table S12 files for demographic details). Alternatively, regular reports about the encroachment of foreign fishers, especially trawlers and artisanal fishers from neighbouring countries [63] as well as destructive fishing practices, such as fish bombing and cyanide fishing [90] potentially causes concern in the communities. Both threats to local fish stocks have received much attention in the popular media and are being discussed repeatedly within communities and among local policy makers [91]. Efforts to collectively mitigate destructive practices such as fish bombing have been made in Maliangin, Banggi Island, Kudat, Berungus Village and Kota Marudu [92]. Upscaling and replication of these community-led marine management techniques remain an area of priority, as identified in the TMP Integrated Management Plan [62]. Increase in fish catch in Kota Marudu is probably owing to increasing fish stocks rather than increasing fishing efforts, given that the number of local artisanal fishers and the type of gears has remained stable. The increase in shellfish aquaculture production in Kudat is likely to be owing to the introduction of white prawn aquaculture since 2014 [93].

Regarding mangroves, local communities in Kudat and Kota Marudu perceived negative trends in mangrove coverage over the last 10 years, although natural science data indicated no or minimal changes. A reverse trend was identified in Pitas, where communities perceived no change in mangrove coverage, whereas the natural science data indicated that mangrove cover has declined. The reason for these divergences might be found in the visibility of parts of the mangrove forests to communities. All coastal communities in Sabah rely on mangrove forests and struggle with their decline owing to the development of shrimp farms, growing human settlements and conversion to agricultural land [94]. It may be that the differences observed between community perceptions and natural science data in Kudat and Kota Marudu reflect a decline in mangrove ecosystem condition which is not reflected in the canopy cover. Given the relatively large mangrove areas of Pitas (150.3 km^2^) compared to those in Kudat (126.0 km^2^) and Kota Marudu (56.8 km^2^) [66], this decline might be less visible for the communities in Pitas.

## 4. Management implications

From the observed patterns of convergence and divergence as well as the site-specific interpretations, we can derive some general patterns leading up to suggestions for successful marine management. We find that divergence might occur for a range of reasons—different data sources working on different levels of specificity, spatial scales or the targeting of different focus areas. It could also be the communities' uncertainty or misconceptions about their available resources. In any case, divergence is a likely source of conflict among resource users or between policy makers and communities [95]. Convergence on the other hand mainly seems to occur together with community-inclusive resource management and a good dialogue between scientists, policy makers and communities. It may also indicate successful existing policies and conservation initiatives [96,97].

The extent of divergence or convergence can inform policy makers or managers of the likelihood that their decisions, especially those based on natural science data, will find acceptance and support among those communities affected by, or implementing the management.

The first suggestion for successful marine management that we identify based on our findings is therefore to support community-inclusive conservation programmes and resource management. We can see a relationship between programmes involving local communities, stakeholders as well as scientists, such as those in Vietnam, Palawan and TMP, and high levels of convergence between what the natural sciences measure and what communities perceive (figure 2). This could be a supportive argument for the co-development of resource management plans as this might create a level of understanding between all involved actors and encourage ownership and responsibility [96,98]. Marine conservation programmes of non-governmental organizations (NGOs), for example, often include end-of-project evaluations that consider ecological changes compared to a baseline. These evaluations are often dependent on sparse natural science data. The here suggested method could supplement this data with people's perceptions and provide a more holistic perspective upon which to evaluate the policy or programme.

The second recommendation is cautious media coverage around ecosystem management including observation and regular evaluation of its effects. Examples from Cu Lao Cham and TMP point towards positive effects of media coverage, potentially contributing to an increase in awareness within the population and supporting sustainable ecosystem management. However, examples from Selayar and Palawan indicate that overly positive media reporting about conservation efforts or beautiful environments does not necessarily preclude effects of ongoing exploitation of natural resources, a lack of implementation of regulation as well as continuing changes owing to climate change and coastal erosion. It is possible that the media's illustration of a pristine island might even lead to neglect to act to prevent continuing decline. It is therefore important to support impartial media reporting, shining light on different perspectives.

An additional recommendation for the interpretation of knowledge integration is to consider community-specific circumstances potentially leading to biases in the reporting of environmental quality trends [53,99]. It is key to understand which role and importance each habitat or ecosystem service has for each community. Further, factors such as tradition, reliance or scarcity can affect their judgement of the habitat [100]. Salient changes in local environments such as loss of mangroves in close versus far distance to the communities can have an impact on community perceptions as illustrated with examples in Taytay and Sabah. Similarly, the (non-) visibility of habitats and their ecosystems or unpopularity in the media can also impact community perceptions as described in the example of seagrass in Vietnam (see also [101]). We therefore recommend considering the limitations and strengths of each data source in relation to the local circumstances.

Lastly, inclusive communication of natural science data trends and associated decisions (e.g. closing down a fishery) is important for community understanding and acceptance [102], as is co-creating datasets in response to local needs and questions. Presenting scientific findings in a discipline-specific, complex matter can trigger feelings of reactance in the audience, and make them feel overwhelmed, unmotivated and helpless [103]. Drawing on evidence from psychology and environmental communication research, tailoring communication can counteract the influence of cognitive biases (for an overview, see [104] or Zhao & Luo [105]). We recommend adapting the communication technique to local preferences and using methods to enhance understanding such as visualizations or maps [106]. Visualizations and maps are useful to illustrate regional differences, to reflect complex interactions or to make invisible resources tangible [107,108]. Another technique, especially for the co-development of resource management plans rather than their evaluation, could be creating future scenarios and thereby outline potential prospects but also encourage community interaction and sustainable engagement [106].

With further development, our suggested methodology could serve as an important platform on which stakeholders may embark on synergistic dialogues [109], especially those involving local communities. Our methodology allows the highlighting of perceptions on ecological trends from a wider population than those few invited to directly participate in marine planning processes. The acknowledgement and incorporation of communities' perspectives regarding their surrounding coastal environments into marine spatial planning are of one of the key aspects to ensure the achievement of UN Sustainable Development Goal (SDG) 14 - life below water [110]. Rather than solely relying on selected natural science data or single consultations to gather stakeholder perspectives, marine planners could directly and visually compare natural science data with social science data over time to feed into marine planning processes, such as the design of marine zones.

## 5. Limitations

The methodology we introduced in this paper comes with a number of limitations.

By their nature, perceptions are subjective and interact with many factors (salience of events, demographics, personal values, habits and more) [56,99]. One could therefore claim that perceptions are not reliable enough for our purpose of informing policymaking or that they cannot be compared with natural science data, discussed in [32]. We suggest that with sample sizes of *n* > 252, representing around 10% of the population in each community (as described in Sumeldan *et al*. [111]), we can assign a quantitative value to community perceptions and define an overall tendency that provides a valid point of comparison with the natural sciences. Owing to economic reasons, our study participants are not fully representative of respective populations (electronic supplementary material, table S12 file for details). We therefore recommend that future research can explore sampling methods with higher levels of demographic representativeness to improve data quality further. Previous studies have also found that local ecological knowledge is often unevenly spread throughout a group and held disproportionately by certain individuals [112]. By creating aggregate scores for all stakeholders, the methodology masks the differences in perspective of different stakeholder groups. Amateur biologists or citizen scientists perspectives may also be of value, e.g. ornithologists historical reflections [113], and such initiatives could be used more systematically within our proposed methodology. In addition, it is important to be transparent about variation in the data by indicating standard deviation or some other suitable indicator.

Another limitation is that we compared not only different traditions of knowledge but that the past and future trends were determined in different ways in natural and social science [24]. In the natural sciences, we combined multiple temporal data points to form a trend line; in the social sciences, we used one data point to represent a trend. The reason for using these different ways of trend representation lies again in the nature of the data and the tradition of knowledge retrieval. We argue that, if interpreted correctly and with awareness of its limitations, both types of knowledge retrieval can be combined, compared and provide useful information. We recommend that research building on our prototype could aspire to collecting longitudinal data in a way that the ecosystem quality is represented by regular assessments of both the natural and the social sciences.

Deviance between knowledge retrieval locations and location size can lead to biases as we discussed across all four sites. Despite the effort to cover areas as similar as possible for both the natural and the social science data, we cannot say with certainty that the communities referred to the exact same spatial sites of study, especially in terms of geographical size. More likely, the natural science data covers a larger area (e.g. the whole park or Biosphere), whereas community members observations are more detailed and situational, and refer to a smaller, individually different area (e.g. their village, fishing ground and immediate surroundings). Similarly, communities may focus their perceptions naturally to accessible areas and areas relevant for their livelihoods, whereas natural science techniques such as remote sensing can produce datasets including non-accessible or remote areas as described in Eisner *et al*. [114]. Tengö *et al*. [10] describe the ‘complementarity' of including data at differing scales, and the benefits of ‘adding in' data from local knowledge. Unsurprisingly, there is limited empirical work that uses both data sources and fully acknowledges and addresses the complications posed. The problem described here cannot fully be solved within this first demonstration of the methodology and must be considered when we interpret our heat maps and followed up with further research. Researchers that aim to develop this methodology further are encouraged to address this limitation. Potential pathways to do this could be to identify how to exactly delineate the areas of origin for both the natural and the social science data or elucidate statistical means to account for the scale of geographical divergence.

A similar limitation, also discussed in Huntington [9] and Tengö *et al*. [10], is the question of comparability when we compare different data sources. While the natural sciences might measure a distinct habitat feature such as the geographical area of mangroves or seagrass in km^2^, communities might refer to ecosystem quality in a more holistic way. As a result, these indicators may diverge but both are valid [115]. In the example of mangroves, remote sensing approaches typically attempt to assess the state of mangroves using estimates of extent [116]. However, previous work has highlighted that area measures alone do not give a complete picture of mangrove condition as they do not provide information on the condition and spatial fragmentation of the habitat that remains [117]. It is important to consider other factors such as changes in the abundance and diversity of fauna, ecosystem functioning and provision of ecosystem services. Communities might report on their impression of general mangrove health, the state of the trees close to their house, intensity of use or the effort of reforestation in their community, which goes beyond the geographical size of a mangrove forest only. This highlights the level of complexity in ecosystem assessments and the dilemma that no single discipline can ever capture the whole picture. Integrating several disciplines can make this picture more complete [10] but still, comparisons between the knowledge sources should always be considered as approximations [24]. For future research, we recommend expanding our methodology and to also integrate quality indicators for both the natural and the social sciences on even more similar levels of specificity.

Diversity of data sources is also problematic. We refer to three different types of fisheries data to assess the trend of wild fish abundance In Malaysia and Indonesia, we used catch data; in the Philippines we used stock assessments. For the future trends, we used modelled outputs of the projected biomass. Each type of data comes with its own limitations. Firstly, accuracy of the catch reporting and recording is not known, and as we do not have the catch effort, we could not normalize the data to catch per unit effort. Secondly, changes in the stock assessment methodology can impact the outcome, with the number and location of the survey sites being one such factor. Finally, the model outputs themselves did not reflect the full fish community but rather a selected number of key species. These outputs are also at a larger spatial scale than catch data or stock assessments. Despite these differences, we suggest that trends of each data type can be used to compare fish data and the community perception data with each other. This is the case as we are applying the same standardized methodology, and specifically because we are comparing the year-to-year change rather than the trend over the full time. This removes extreme values coming from the landing or assessment data, as well as the spatial bias from the coarse resolution in future models. Using different types of data for a similar concept, the abundance of wild fishes, demonstrates how versatile and adaptive our methodology is.

## 6. Conclusion

Finding ways to systematically combine ecosystem data from different knowledge systems is an important and challenging step towards inclusive and collaborative resource management and for gaining constructive insights into trends in habitats and ecosystems over time. In this work we have demonstrated a standardized methodology for data integration, providing information about convergence and divergence of social and natural science data regarding past and future trends in ecosystem quality. This prototype of our methodology responds to the UN Ocean Decade's call for global collaboration and localized solutions, interdisciplinary exchange of knowledge and integrative methodologies. The data from Vietnam, the Philippines, Malaysia and Indonesia integrated in this work was selected owing to its local relevance and the availability within the GCRF Blue Communities project. We would like to emphasize that the approach can and should be extended, advanced and applied to other types of disciplines, datasets and regions.

The four regional discussions are an illustration of how this data can shed light on the effectiveness of current regulations but also inform and shape future effective resource management and accompanying communication. From our site-specific discussion, we have formulated four key recommendations for successful resource management using different data sources, which are: (i) community-inclusive development and implementation of regulations, (ii) well-informed reporting by mass-media and dissemination to different stakeholders, (iii) awareness and consideration of data-specific biases, and (iv) tailored communication through visualizations, maps and future scenarios. The different profiles of convergence and divergence between our included sites also underline the value of localized resource management and climate change responses as recently stated at the Conference of Parties (COP26) in Glasgow [118].

We advocate that, in addition to generating new scientific knowledge for marine resource conservation, we need to nurture the art of bringing this knowledge together, inclusively and in an accessible format and thereby provide ‘*The science we need for the ocean we want*’ [2].

## Data Availability

Data can be found on Zenodo: https://doi.org/10.5281/zenodo.6378743. The data are provided in the electronic supplementary material [119].

## References

[1] Claudet J, Bopp L, Cheung WWL, Devillers R, Escobar-Briones E, Haugan P, Gaill F. 2020 A roadmap for using the un decade of ocean science for sustainable development in support of science, policy, and action. One Earth **2**, 34-42. (10.1016/j.oneear.2019.10.012)

[2] United Nations. 2020 *The science we need for the ocean we want: the United Nations Decade of Ocean Science for Sustainable Development (2021–2030)*. Paris, France: United Nations.

[3] Burdon D, Boyes SJ, Elliott M, Smyth K, Atkins JP, Barnes RA, Wurzel RK. 2018 Integrating natural and social sciences to manage sustainably vectors of change in the marine environment: Dogger Bank transnational case study. Estuarine Coastal Shelf Sci. **201**, 234-247. (10.1016/j.ecss.2015.09.012)

[4] Bennett NJ, Di Franco A, Calò A, Nethery E, Niccolini F, Milazzo M, Guidetti P. 2019 Local support for conservation is associated with perceptions of good governance, social impacts, and ecological effectiveness. Conserv. Lett. **12**, e12640. (10.1111/conl.12640)

[5] Flannery W, Toonen H, Jay S, Vince J. 2020 A critical turn in marine spatial planning. Maritime Stud. **19**, 223-228. (10.1007/s40152-020-00198-8) PMC7447205 38624457

[6] Reid WV, Chen D, Goldfarb L, Hackmann H, Lee YT, Mokhele K, Whyte A. 2010 Earth system science for global sustainability: grand challenges. Science **330**, 916-917. (10.1126/science.1196263) 21071651

[7] Pomeroy R, Douvere F. 2008 The engagement of stakeholders in the marine spatial planning process. Mar. Policy **32**, 816-822. (10.1016/j.marpol.2008.03.017)

[8] Belhabib D, Le Billon P, Bennett NJ. 2021 Ocean sustainability for all requires deeper behavioural research. Nat. Hum. Behav. **6**, 6-8. (10.1038/s41562-021-01256-9) 34857926

[9] Huntington HP. 2000 Using traditional ecological knowledge in science: methods and applications. Ecol. Appl. **10**, 1270-1274. (10.1890/1051-0761(2000)010[1270:UTEKIS]2.0.CO;2)

[10] Tengö M, Brondizio ES, Elmqvist T, Malmer P, Spierenburg M. 2014 Connecting diverse knowledge systems for enhanced ecosystem governance: the multiple evidence base approach. Ambio **43**, 579-591. (10.1007/s13280-014-0501-3) 24659474 PMC4132468

[11] O'Brien CM, Fox CJ, Planque B, Casey J. 2000 Climate variability and North Sea cod. Nature **404**, 142. (10.1038/35004654) 10724155

[12] Jung CA, Dwyer PD, Minnegal M, Swearer SE. 2011 Perceptions of environmental change over more than six decades in two groups of people interacting with the environment of Port Phillip Bay, Australia. Ocean Coastal Manag. **54**, 93-99. (10.1016/j.ocecoaman.2010.10.035)

[13] Parsons DM, Morrison MA, MacDiarmid AB, Stirling B, Cleaver P, Smith IWG, Butcher M. 2009 Risks of shifting baselines highlighted by anecdotal accounts of New Zealand's snapper (*Pagrus auratus*) fishery. New Zealand J. Mar. Freshwater Res. **43**, 965-983. (10.1080/00288330909510054)

[14] Tin HC, Ni TNK, Tuan LV, Saizen I, Catherman R. 2019 Spatial and temporal variability of mangrove ecosystems in the Cu Lao Cham-Hoi An Biosphere Reserve, Vietnam. Regional Stud. Mar. Sci. **27**, 100550. (10.1016/j.rsma.2019.100550)

[15] Tin HC, Uyen NT, Hieu DV, Ni TNK, Tu NHC, Saizen I. 2020 Decadal dynamics and challenges for seagrass beds management in Cu Lao Cham Marine Protected Area, Central Vietnam. Environ. Dev. Sustain. **22**, 7639-7660. (10.1007/s10668-019-00540-z)

[16] Torrents-Ticó M, Fernández-Llamazares Á, Burgas D, Cabeza M. 2021 Convergences and divergences between scientific and indigenous and local knowledge contribute to inform carnivore conservation. Ambio **50**, 990-1002. (10.1007/s13280-020-01443-4) 33438166 PMC8035381

[17] Laidler GJ. 2006 Inuit and scientific perspectives on the relationship between sea ice and climate change: the ideal complement? Clim. Change **78**, 407. (10.1007/s10584-006-9064-z)

[18] Moller H, Berkes F, Lyver POB, Kislalioglu M. 2004 Combining science and traditional ecological knowledge: monitoring populations for co-management. Ecol. Soc. **9**, 2. (10.5751/ES-00675-090302)

[19] Mackinson S. 2001 Integrating local and scientific knowledge: an example in fisheries science. Environ. Manage. **27**, 533-545. (10.1007/s0026702366) 11289452

[20] Bennett NJ. 2016 Using perceptions as evidence to improve conservation and environmental management. Conserv. Biol. **30**, 582-592. (10.1111/cobi.12681) 26801337

[21] Le Cornu E, Kittinger JN, Koehn JZ, Finkbeiner EM, Crowder LB. 2014 Current practice and future prospects for social data in coastal and ocean planning. Conserv. Biol. **28**, 902-911. (10.1111/cobi.12310) 24779578

[22] O'Leary BC et al. 2021 The nature and extent of evidence on methodologies for monitoring and evaluating marine spatial management measures in the UK and similar coastal waters: a systematic map. Environment. Evidence **10**, 13. (10.1186/s13750-021-00227-x)

[23] Tengö M, Hill R, Malmer P, Raymond CM, Spierenburg M, Danielsen F, Folke C. 2017 Weaving knowledge systems in IPBES, CBD and beyond—lessons learned for sustainability. Curr. Opin. Environ. Sustain. **26**, 17-25. (10.1016/j.cosust.2016.12.005)

[24] Bohensky E, Maru Y. 2011 Indigenous knowledge, science, and resilience: what have we learned from a decade of international literature on ‘integration’? Ecol. Soc. **16**, 6. (10.5751/ES-04342-160406)

[25] Whyte KP, Brewer JP, Johnson JT. 2016 Weaving Indigenous science, protocols and sustainability science. Sustainability Sci. **11**, 25-32. (10.1007/s11625-015-0296-6)

[26] Berkes F, Berkes MK. 2009 Ecological complexity, fuzzy logic, and holism in indigenous knowledge. Futures **41**, 6-12. (10.1016/j.futures.2008.07.003)

[27] Kutz S, Tomaselli M. 2019 ‘Two-eyed seeing’ supports wildlife health. Science **364**, 1135-1137. (10.1126/science.aau6170) 31221846

[28] Armitage D, Berkes F, Doubleday N. 2010 Adaptive co-management: collaboration, learning, and multi-level governance. Vancouver, Canada: UBC Press.

[29] Cottet M, Piégay H, Bornette G. 2013 Does human perception of wetland aesthetics and healthiness relate to ecological functioning? J. Environ. Manage. **128**, 1012-1022. (10.1016/j.jenvman.2013.06.056) 23895913

[30] Ioana-Toroimac G, Zaharia L, Neculau G, Constantin DM, Stan FI. 2020 Translating a river's ecological quality in ecosystem services: an example of public perception in Romania. Ecohydrol. Hydrobiol. **20**, 31-37. (10.1016/j.ecohyd.2019.10.005)

[31] Foale S. 2006 The intersection of scientific and indigenous ecological knowledge in coastal Melanesia: implications for contemporary marine resource management. Int. Soc. Sci. J. **58**, 129-137. (10.1111/j.1468-2451.2006.00607.x)

[32] Berkes F. 1993 Traditional ecological knowledge in perspective. University of Oregon, Eugene, OR: Canadian Museum of Nature/International Development Research Centre.

[33] Jefferson R, McKinley E, Griffin H, Nimmo A, Fletcher S. 2021 Public perceptions of the ocean: lessons for marine conservation from a global research review. Front. Mar. Sci. **8**, 711245. (10.3389/fmars.2021.711245)

[34] Gifford R, Scannell L, Kormos C, Smolova L, Biel A, Boncu S, Hine D. 2009 Temporal pessimism and spatial optimism in environmental assessments: An 18-nation study. J. Environ. Psychol. **29**, 1-12. (10.1016/j.jenvp.2008.06.001)

[35] Fincher R, Barnett J, Graham S. 2015 Temporalities in adaptation to sea-level rise. Ann. Assoc. Amer. Geogr. **105**, 263-273. (10.1080/00045608.2014.988101)

[36] Papworth S, Rist J, Coad L, Milner-Gulland E. 2009 Evidence for shifting baseline syndrome in conservation. *Conserv. Lett*. **2**, 93-100. (10.1111/j.1755-263X.2009.00049.x)

[37] Flotemersch J, Aho K. 2021 Factors influencing perceptions of aquatic ecosystems. Ambio **50**, 425-435. (10.1007/s13280-020-01358-0) 32700206 PMC7782621

[38] Doney SC, Sailley SF. 2013 When an ecological regime shift is really just stochastic noise. Proc. Natl Acad. Sci. USA **110**, 2438-2439. (10.1073/pnas.1222736110) 23382242 PMC3574937

[39] Hsieh C-h, Glaser SM, Lucas AJ, Sugihara G. 2005 Distinguishing random environmental fluctuations from ecological catastrophes for the North Pacific Ocean. Nature **435**, 336-340. (10.1038/nature03553) 15902256

[40] Powney GD, Isaac NJB. 2015 Beyond maps: a review of the applications of biological records. Biol. J. Linnean Soc. **115**, 532-542. (10.1111/bij.12517)

[41] Isaac NJB, Pocock MJO. 2015 Bias and information in biological records. Biol. J. Linnean Soc. **115**, 522-531. (10.1111/bij.12532)

[42] Johannes RE, Freeman MMR, Hamilton RJ. 2000 Ignore fishers' knowledge and miss the boat. Fish and Fisheries **1**, 257-271. (10.1111/j.1467-2979.2000.00019.x)

[43] Hedley JD, Roelfsema CM, Chollett I, Harborne AR, Heron SF, Weeks S, Mumby PJ. 2016 Remote sensing of coral reefs for monitoring and management: a review. Remote Sensing **8**, 118. (10.3390/rs8020118)

[44] Hashioka T, Vogt M, Yamanaka Y, Le Quéré C, Buitenhuis ET, Aita MN, Doney SC. 2013 Phytoplankton competition during the spring bloom in four plankton functional type models. Biogeosciences **10**, 6833-6850. (10.5194/bg-10-6833-2013)

[45] Maar M, Butenschön M, Daewel U, Eggert A, Fan W, Hjøllo SS, van de Wolfshaar K. 2018 Responses of summer phytoplankton biomass to changes in top-down forcing: insights from comparative modelling. Ecol. Model. **376**, 54-67. (10.1016/j.ecolmodel.2018.03.003)

[46] Sailley SF, Vogt M, Doney SC, Aita MN, Bopp L, Buitenhuis ET, Yamanaka Y. 2013 Comparing food web structures and dynamics across a suite of global marine ecosystem models. Ecol. Model. **261–262**, 43-57. (10.1016/j.ecolmodel.2013.04.006)

[47] Eyring V, Bony S, Meehl GA, Senior CA, Stevens B, Stouffer RJ, Taylor KE. 2016 Overview of the Coupled Model Intercomparison Project Phase 6 (CMIP6) experimental design and organization. Geosci. Model Dev. **9**, 1937-1958. (10.5194/gmd-9-1937-2016)

[48] Lotze HK, Tittensor DP, Bryndum-Buchholz A, Eddy TD, Cheung WWL, Galbraith ED, Worm B. 2019 Global ensemble projections reveal trophic amplification of ocean biomass declines with climate change. Proc. Natl Acad. Sci. USA **116**, 12 907-12 912. (10.1073/pnas.1900194116) PMC6600926 31186360

[49] Spitz YH, Moisan JR, Abbott MR, Richman JG. 1998 Data assimilation and a pelagic ecosystem model: parameterization using time series observations. J. Mar. Sys. **16**, 51-68. (10.1016/S0924-7963(97)00099-7)

[50] Sailley SF, Ducklow HW, Moeller HV, Fraser WR, Schofield OM, Steinberg DK, Doney SC. 2013 Carbon fluxes and pelagic ecosystem dynamics near two western Antarctic Peninsula Adélie penguin colonies: an inverse model approach. Mar. Ecol. Progress Series **492**, 253-272. (10.3354/meps10534)

[51] Skogen MD, Ji R, Akimova A, Daewel U, Hansen C, Hjøllo SS, van de Wolfshaar K. 2021 Disclosing the truth: are models better than observations? Mar. Ecol. Progress Series **680**, 7-13. See https://www.int-res.com/abstracts/MEPS/dynmod/p_av1.

[52] Bell RJ, McManus MC, McNamee J, Gartland J, Galuardi B, McGuire C. 2021 Perspectives from the water: utilizing fisher's observations to inform SNE/MA windowpane science and management. Fish. Res. **243**, 106090. (10.1016/j.fishres.2021.106090)

[53] Griffin L. 2009 Scales of knowledge: North Sea fisheries governance, the local fisherman and the European scientist. Environ. Politics **18**, 557-575. (10.1080/09644010903007419)

[54] Huntington HP. 2011 The local perspective. Nature **478**, 182-183. (10.1038/478182a) 21993743

[55] Agrawal A. 1995 Dismantling the divide between indigenous and scientific knowledge. Dev. Change **26**, 413-439. (10.1111/j.1467-7660.1995.tb00560.x)

[56] Forsyth T. 2004 Critical political ecology: the politics of environmental science. Abingdon, UK: Routledge.

[57] Hill R, Adem Ç, Alangui WV, Molnár Z, Aumeeruddy-Thomas Y, Bridgewater P, Xue D. 2020 Working with Indigenous, local and scientific knowledge in assessments of nature and nature's linkages with people. Curr. Opin. Environ. Sustain. **43**, 8-20. (10.1016/j.cosust.2019.12.006)

[58] Ngoc QTK. 2018 Impacts on the ecosystem and human well-being of the marine protected area in Cu Lao Cham, Vietnam. Mar. Policy **90**, 174-183. (10.1016/j.marpol.2017.12.015)

[59] PCSD. 2015 *State of the Environment 2015 Updates, Province of Palawan (UNESCO Man and Biosphere Reserve)*. Retrieved from Puerto Princesa City, Philippines.

[60] Selayar Statistical Bureau. 2019 *Kabupaten Kepulauan Selayar Dalam Angka*. See https://selayarkab.bps.go.id/publication/download.html?nrbvfeve=ZmNkNTU4YmIyNTY4YmFjYzY5MGNkNmRi&xzmn=aHR0cHM6Ly9zZWxheWFya2FiLmJwcy5nby5pZC9wdWJsaWNhdGlvbi8yMDE5LzA4LzE2L2ZjZDU1OGJiMjU2OGJhY2M2OTBjZDZkYi9rYWJ1cGF0ZW4ta2VwdWxhdWFuLXNlbGF5YXItZGFsYW0tYW5na2EtMjAxOS5odG1s&twoadfnoarfeauf=MjAyMS0wNi0zMCAyMDo0NzozMw%3D%3D.

[61] UNESCO. 2015 *Taka Bonerate Kepulauan Selayar*. See http://www.unesco.org/new/en/natural-sciences/environment/ecological-sciences/biosphere-reserves/asia-and-the-pacific/indonesia/taka-bonerate-kepulauan-selayar.

[62] Sabah Parks. 2017 *Tun Mustapha Park Integrated Management Plan (2017–2026)*. Retrieved from Kota Kinabalu, Malaysia.

[63] Teh L, Teh L, Sumaila UR. 2011 Quantifying the overlooked socio-economic contribution of small-scale fisheries in Sabah, Malaysia. Fish. Res. **110**, 450-458. (10.1016/j.fishres.2011.06.001)

[64] Cheung V et al. 2021 *Blue Communities in Southeast Asia*. See https://www.the-ies.org/sites/default/files/journals/sustainable-future-ocean-decade.pdf.

[65] Teh L, Cabanban AS, Sumaila UR. 2005 The reef fisheries of Pulau Banggi, Sabah: a preliminary profile and assessment of ecological and socio-economic sustainability. Fish. Res. **76**, 359-367. (10.1016/j.fishres.2005.07.009)

[66] Bunting P, Rosenqvist A, Lucas RM, Rebelo L-M, Hilarides L, Thomas N, Finlayson CM. 2018 The Global Mangrove Watch—a new 2010 global baseline of mangrove extent. Remote Sensing **10**, 1669. (10.3390/rs10101669)

[67] Goldberg L, Lagomasino D, Thomas N, Fatoyinbo T. 2020 Global declines in human-driven mangrove loss. Glob. Change Biol. **26**, 5844-5855. (10.1111/gcb.15275) PMC7540710 32654309

[68] Richards DR, Friess DA. 2016 Rates and drivers of mangrove deforestation in Southeast Asia, 2000–2012. Proc. Natl Acad. Sci. USA **113**, 344-349. (10.1073/pnas.1510272113) 26712025 PMC4720307

[69] PPCAO. 2017 *Compilation of Reports on Coastal Resource Assessment in Puerto Princesa City*. Retrieved from Puerto Princesa City, Philippines.

[70] English S, Wilkinson C, Baker V. 1997 *Survey manual for tropical marine resources*, 2nd edn. Townsville, Australia: ASEAN-Australia Marine Science Project: Living Coastal Resources, Australian Institute of Marine Science.

[71] Uychiangco AJ, Green JS, dela Cruz MT, Gaite PA, Arceo HO, Alino PM, White AT. 2001 Coral reef monitoring for management. Palawan, Philippines: GDFI, Voluntary Service Overseas, UP-CIDS, CRMP and FRMP.

[72] Hodgson G, Hill J, Kiene W, Maun L, Mihaly J, Liebeler J, Torres R. 2006 Instruction manual. A guide to coral reef monitoring. Pacific Palisades, CA: Reef Check Foundation.

[73] Richards JA. 2006 Remote sensing digital image analysis, vol. 3, Berlin, Germany: Springer.

[74] Green E, Mumby P, Edwards A, Clark C. 2000 Remote sensing: handbook for tropical coastal management. Paris, France: United Nations Educational, Scientific and Cultural Organization (UNESCO).

[75] Bruno JF, Selig ER. 2007 Regional decline of coral cover in the Indo-Pacific: timing, extent, and subregional comparisons. PLoS ONE **2**, e711. (10.1371/journal.pone.0000711) 17684557 PMC1933595

[76] Waycott M, Duarte CM, Carruthers TJB, Orth RJ, Dennison WC, Olyarnik S, Williams SL. 2009 Accelerating loss of seagrasses across the globe threatens coastal ecosystems. Proc. Natl Acad. Sci. USA **106**, 12 377-12 381. (10.1073/pnas.0905620106) 19587236 PMC2707273

[77] Sudo K, Quiros TEAL, Prathep A, Luong CV, Lin H-J, Bujang JS, Nakaoka M. 2021 Distribution, temporal change, and conservation status of tropical seagrass beds in Southeast Asia: 2000–2020. Front. Mar. Sci. **8**, 1-11. (10.3389/fmars.2021.637722)

[78] Bulengela G, Onyango P, Brehm J, Staehr PA, Sweke E. 2020 ‘Bring fishermen at the center’: the value of local knowledge for understanding fisheries resources and climate-related changes in Lake Tanganyika. Environ. Dev. Sust. **22**, 5621-5649. (10.1007/s10668-019-00443-z)

[79] SGPG, UNDP. 2018 Rehabilitation and conservation of Nyapalms at Cam Thanh Commune, Hoi An city, Quang Nam. See https://www.vn.undp.org/content/vietnam/en/home/library/environment_climate/rehabilitation-and-conservation-of-nyapalms-at-cam-thanh-commune.html.

[80] Primavera JH. 2004 Philippine mangroves: status, threats, and sustainable development. In Mangrove management and conservation: present and future (ed. M Vannucci), pp. 192-207. Tokyo, Japan: United Nations University Press.

[81] CCRES. 2014 Capturing coral reef and related ecosystem services. See https://ccres.net/about/about-ccres.

[82] Coremap. 2016 Coremap Phase 1. See http://coremap.oseanografi.lipi.go.id/berita/15#.

[83] Lampe M. 2017 *Coral Reef Fisheries Resource Management in Taka Bonerate National Park Based on Constructionism Perspective BT.* Paper presented at the Proceedings of the Unhas International Conference on Social and Political Science (UICoSP 2017).

[84] Praptiwi RA, Maharja C, Fortnam M, Chaigneau T, Evans L, Garniati L, Sugardjito J. 2021 Tourism-based alternative livelihoods for small island communities transitioning towards a blue economy. Sustainability **13**, 6655. (10.3390/su13126655)

[85] Kusumo S, Adrianto L, Boer M. 2020 A system dynamics model for marine conservation area management–a case study of Pulo Pasi Gusung local marine conservation area, Selayar, Indonesia. Aquacult. Aquarium Conserv. Legislation **13**, 715-735. See http://www.bioflux.com.ro/aacl.

[86] Moore AM, Ambo-Rappe R, Ali Y. 2017 ‘The lost princess (putri duyung)’ of the small islands: dugongs around Sulawesi in the anthropocene. Front. Mar. Sci. **4**, 284. (10.3389/fmars.2017.00284)

[87] Simmons EC, Fielding KS. 2019 Psychological predictors of fishing and waste management intentions in Indonesian coastal communities. J. Environ. Psychol. **65**, 101324. (10.1016/j.jenvp.2019.101324)

[88] Rothschild ZK, Landau MJ, Sullivan D, Keefer LA. 2012 A dual-motive model of scapegoating: displacing blame to reduce guilt or increase control. J. Pers. Soc. Psychol. **102**, 1148-1163. (10.1037/a0027413) 22545745

[89] Ainsworth CH, Pitcher TJ, Rotinsulu C. 2008 Evidence of fishery depletions and shifting cognitive baselines in Eastern Indonesia. Biol. Conserv. **141**, 848-859. (10.1016/j.biocon.2008.01.006)

[90] Biusing ER 2001 *Assessment of Coastal Fisheries in the Malaysian- Sabah portion of the Sulu-Sulawesi Marine Ecoregion (SSME)*. Retrieved from Sabah, Malaysia.

[91] Cooke FM. 2003 *Living at the top end: communities and natural resource use in the Kudat/Banggi region of Northern Sabah, Malaysia*.

[92] Wildaid. 2017 *The Tun Mustapha Compliance Plan*. Retrieved from San Francisco, California, USA: https://wildaid.org/wp-content/uploads/2017/09/The-Tun-Mustapha-Compliance-Plan-2017.pdf.

[93] Department of Fisheries Sabah. 2018 *Annual Fisheries Statistics: 2009–2018*. Retrieved from Malaysia, see https://fishdept.sabah.gov.my/?q=en/fisheries-statistics.

[94] Mojiol AR, Ismenyah M, Lintangah WJ, Pendrongi B, Wahyudi. 2017 Mangrove forest in Kudat, Sabah Malaysia: challenges of the mangrove conservation. Jurnal Hutan Tropika **12**, 1-12.

[95] Neis B, Schneider DC, Felt L, Haedrich RL, Fischer J, Hutchings JA. 1999 Fisheries assessment: what can be learned from interviewing resource users? Can. J. Fish. Aquat. Sci. **56**, 1949-1963. (10.1139/f99-115)

[96] Hamilton RJ, Giningele M, Aswani S, Ecochard JL. 2012 Fishing in the dark-local knowledge, night spearfishing and spawning aggregations in the Western Solomon Islands. Biol. Conserv. **145**, 246-257. (10.1016/j.biocon.2011.11.020)

[97] Stephenson J, Moller H. 2009 Cross-cultural environmental research and management: challenges and progress. J. R. Soc. New Zealand **39**, 139-149. (10.1080/03014220909510567)

[98] Butler JRA, Suadnya W, Yanuartati Y, Meharg S, Wise RM, Sutaryono Y, Duggan K. 2016 Priming adaptation pathways through adaptive co-management: design and evaluation for developing countries. Climate Risk Management **12**, 1-16. (10.1016/j.crm.2016.01.001)

[99] Verweij MC, van Densen WLT, Mol AJP. 2010 The tower of Babel: different perceptions and controversies on change and status of North Sea fish stocks in multi-stakeholder settings. Mar. Policy **34**, 522-533. See https://EconPapers.repec.org/RePEc:eee:marpol:v:34:y:2010:i:3:p:522–533. (10.1016/j.marpol.2009.10.008)

[100] Lau JD, Hicks CC, Gurney GG, Cinner JE. 2019 What matters to whom and why? Understanding the importance of coastal ecosystem services in developing coastal communities. Ecosystem Services **35**, 219-230. (10.1016/j.ecoser.2018.12.012)

[101] McKinley E, Pagès JF, Ballinger RC, Beaumont N. 2020. Forgotten landscapes: public attitudes and perceptions of coastal saltmarshes. *Ocean Coastal Manage*. **187**, 105117.

[102] Maurstad A. 2002 Fishing in murky waters—ethics and politics of research on fisher knowledge. Marine Policy **26**, 159-166. (10.1016/S0308-597X(01)00045-8)

[103] Marshall G. 2015 Don't even think about it: why our brains are wired to ignore climate change. London, UK: Bloomsbury Publishing.

[104] Richter I, Gabe-Thomas E, Queirós AM, Sheppard S, Pahl S. In preparation. Maximising the potential of future scenarios by integrating psychological principles. *Environ. Sci. Policy*.

[105] Zhao J, Luo Y. 2021 A framework to address cognitive biases of climate change. Neuron **109**, 3548-3551. (10.1016/j.neuron.2021.08.029) 34555315

[106] Richter I, Sumeldan J, Avillanosa A, Gabe-Thomas E, Creencia L, Pahl S. 2021 Co-created future scenarios as a tool to communicate sustainable development in coastal communities in Palawan, Philippines. Front. Psychol. **12**, 627972. (10.3389/fpsyg.2021.627972) 34880799 PMC8645572

[107] Fischer H, van den Broek KL, Ramisch K, Okan Y. 2020 When IPCC graphs can foster or bias understanding: evidence among decision-makers from governmental and non-governmental institutions. Environ. Res. Lett. **15**, 114041. (10.1088/1748-9326/abbc3c)

[108] Sheppard S. 2012 Visualizing climate change: a guide to visual communication of climate change and developing local solutions. Abingdon, UK: Routledge.

[109] Bakker YW, de Koning J, van Tatenhove J. 2019 Resilience and social capital: the engagement of fisheries communities in marine spatial planning. Mar. Policy **99**, 132-139. (10.1016/j.marpol.2018.09.032)

[110] Ntona M, Morgera E. 2018 Connecting SDG 14 with the other Sustainable Development Goals through marine spatial planning. Mar. Policy **93**, 214-222. (10.1016/j.marpol.2017.06.020)

[111] Sumeldan J, Richter I, Avillanosa A, Bacosa H, Creencia L, Pahl S. 2021 Ask the locals: a community-informed analysis of perceived marine environment quality over time in Palawan, Philippines. Front. Psychol.– Environ. Psychol. **12**, 661810. (10.3389/fpsyg.2021.661810) PMC8382879 34447327

[112] Crona B, Bodin O. 2006 What you know is who you know? Communication patterns among resource users as a prerequisite for co-management. Ecol. Soc. **11**, 7. See http://www.ecologyandsociety.org/vol11/iss2/art7/.

[113] Reif J, Szarvas F, Šťastný K. 2021 ‘Tell me where the birds have gone’ – Reconstructing historical influence of major environmental drivers on bird populations from memories of ornithologists of an older generation. Ecol. Indic. **129**, 107909. (10.1016/j.ecolind.2021.107909)

[114] Eisner WR, Cuomo CJ, Hinkel KM, Jones BM, Brower RH. 2009 Advancing landscape change research through the incorporation of Iñupiaq knowledge. Arctic **62**, 429-442. (10.14430/arctic174)

[115] Conchedda G, Lambin EF, Mayaux P. 2011 Between land and sea: livelihoods and environmental changes in mangrove ecosystems of Senegal. Ann. Assoc. Am. Geograph. **101**, 1259-1284. (10.1080/00045608.2011.579534)

[116] Friess DA, Rogers K, Lovelock CE, Krauss KW, Hamilton SE, Lee SY, Shi S. 2019 The state of the world's mangrove forests: past, present, and future. Annu. Rev. Environ. Resour. **44**, 89-115. (10.1146/annurev-environ-101718-033302)

[117] Bryan-Brown DN, Connolly RM, Richards DR, Adame F, Friess DA, Brown CJ. 2020 Global trends in mangrove forest fragmentation. Sci. Rep. **10**, 7117. (10.1038/s41598-020-63880-1) 32346000 PMC7188678

[118] SDG Knowledge Hub. 2021 Vast majority of climate actions must be local: COP 26 Pavilion Discussions (Press release). See http://sdg.iisd.org/news/vast-majority-of-climate-actions-must-be-local-cop-26-pavilion-discussions/?utm_medium=email&utm_campaign=SDG%20Weekly%20Update%20-%2019%20November%202021&utm_content=SDG%20Weekly%20Update%20-%2019%20November%202021+CID_f301394adfaacc9916061706170d890d&utm_source=cm&utm_term=Read.

[119] Richter I et al. 2022 Data from: Building bridges between natural and social science disciplines: a standardized methodology to combine data on ecosystem quality trends. *Figshare*. (10.6084/m9.figshare.c.5962318) PMC9108946 35574850

